# Spatial Arrangement and Set Size Influence the Coding of Non-symbolic Quantities in the Intraparietal Sulcus

**DOI:** 10.3389/fnhum.2018.00054

**Published:** 2018-02-20

**Authors:** Johannes Bloechle, Julia F. Huber, Elise Klein, Julia Bahnmueller, Johannes Rennig, Korbinian Moeller, Stefan Huber

**Affiliations:** ^1^Neurocognition Lab, Leibniz-Institut für Wissensmedien, Knowledge Media Research Center, Tübingen, Germany; ^2^Division of Neuropsychology, Center of Neurology, Hertie-Institute for Clinical Brain Research, University of Tübingen, Tübingen, Germany; ^3^Department of Psychology, University of Tübingen, Tübingen, Germany; ^4^Department of Neurosurgery, Baylor College of Medicine, Houston, TX, United States; ^5^LEAD Graduiertenschule und Forschungsnetzwerk, Universität Tübingen, Tübingen, Germany

**Keywords:** IPS, subitizing, non-symbolic magnitude, numerical cognition, approximate number system

## Abstract

Performance in visual quantification tasks shows two characteristic patterns as a function of set size. A precise subitizing process for small sets (up to four) was contrasted with an approximate estimation process for larger sets. The spatial arrangement of elements in a set also influences visual quantification performance, with frequently perceived arrangements (e.g., dice patterns) being faster enumerated than random arrangements. Neuropsychological and imaging studies identified the intraparietal sulcus (IPS), as key brain area for quantification, both within and above the subitizing range. However, it is not yet clear if and how set size and spatial arrangement of elements in a set modulate IPS activity during quantification. In an fMRI study, participants enumerated briefly presented dot patterns with random, canonical or dice arrangement within and above the subitizing range. We evaluated how activity amplitude and pattern in the IPS were influenced by size and spatial arrangement of a set. We found a discontinuity in the amplitude of IPS response between subitizing and estimation range, with steep activity increase for sets exceeding four elements. In the estimation range, random dot arrangements elicited stronger IPS response than canonical arrangements which in turn elicited stronger response than dice arrangements. Furthermore, IPS activity patterns differed systematically between arrangements. We found a signature in the IPS response for a transition between subitizing and estimation processes during quantification. Differences in amplitude and pattern of IPS activity for different spatial arrangements indicated a more precise representation of non-symbolic numerical magnitude for dice and canonical than for random arrangements. These findings challenge the idea of an abstract coding of numerosity in the IPS even within a single notation.

## Introduction

The ability to discriminate sets of entities based on their number is one of the most basic numerical competencies. Infants already show it prior to the emergence of language and symbolic counting. When presented with arrays of dots, sequences of sounds or tactile stimulations, even 6 month olds are able to discriminate between numerosities (Starkey and Cooper, 1980; Xu and Spelke, 2000; Xu et al., 2005). However, this remarkable sensitivity to numerosity is not an exclusively human quality. Comparative psychology demonstrated basic quantification skills in a wide variety of species, ranging from honey bees over fish to monkeys and great apes (Hanus and Call, 2007; Beran, 2008; Dacke and Srinivasan, 2008; Agrillo et al., 2012). Collectively, these findings provide support for the existence of an evolutionarily ancient cognitive quantification system that enables human and non-human animals to estimate and compare numerosities without counting verbally (Dehaene, 2001).

Electrophysiological studies in monkeys provide insights into the underlying mechanisms of this system, which is often referred to as approximate number system or approximate number sense (ANS; Feigenson et al., 2004; Cantlon et al., 2009; Piazza, 2010). Researchers demonstrated that specific neurons in the lateral prefrontal cortex and the intraparietal sulcus (IPS) responded maximally to specific numerosities (e.g., the number of dots in visual multiple-dot displays; Nieder et al., 2002; Nieder, 2005, 2012; see Ditz and Nieder, 2015 for similar findings in crows). Although these numerosity selective neurons were tuned to preferred numerosities, their spike rate also increased when adjacent numerosities were presented. This led to the idea that numerosity is represented in form of overlapping, bell-shaped (Gaussian) tuning curves which increase in width (i.e., imprecision) with increasing numerosity (for reviews see Nieder and Dehaene, 2009; Nieder, 2016). Human neuroimaging studies provided further support for the existence of a quantification system with numerosity selective neurons as neuronal underpinning (Nieder and Dehaene, 2009; Lyons et al., 2015). For instance, by using a representational similarity approach Lyons et al. (2015) suggested that non-symbolic numerosities in humans are indeed represented in bilateral IPS by overlapping bell-shaped tuning curves with increasing neuronal overlap with increasing numerosities.

Psychophysical studies provided further evidence for a qualitative distinction between quantification of large and small visual arrays. For small set sizes containing up to four elements, numerosity judgments were observed to be precise, effortless, and extremely rapid. However, when a set exceeded four items, a distinctive discontinuity in the slopes of response speed and accuracy was observed (Jevons, 1871; Kaufman et al., 1949; Trick and Pylyshyn, 1994). The fast, accurate, and non-verbal quantification process of small set sizes up to four elements was coined “subitizing” (Kaufman et al., 1949; Mandler and Shebo, 1982). In contrast, quantification of larger set sizes under time restriction that prevents serial counting is assumed to involve an approximate estimation process (Feigenson et al., 2004; Revkin et al., 2008).

Neuropsychological and neuroimaging studies indicate that the parietal cortex, in particular the intraparietal sulcus, is a key brain area for visual quantification processes both within *and* above the subitizing range (Piazza et al., 2002, 2004; Dehaene et al., 2003; Demeyere et al., 2014). An fMRI adaptation study identified bilateral IPS as the only brain region sensitive to a change in numerosity of dot patterns in a passive viewing task (Piazza et al., 2004). Recently, Demeyere et al. (2014) suggested that this critical involvement of the IPS does not depend on set size, since increased IPS activity was observed during quantification of small and large sets of dots. In this line, using functional near-infrared spectroscopy (fNIRS) Cutini et al. (2014) observed hemodynamic activity in the IPS during dot pattern quantification both in the subitizing and the estimation range. Neuropsychological case studies reported severe impairments in quantification performance in both subitizing and estimation range for patients with lesions affecting the IPS (Lemer et al., 2003; Ashkenazi et al., 2008). Supporting these findings, developmental dyscalculia, for which impaired subitizing and estimation performance was reported (e.g., Ashkenazi et al., 2013), was associated with a reduction of gray matter volume in the left and right IPS (Isaacs et al., 2001; Rotzer et al., 2008).

Visual quantification performance is also affected by the spatial arrangement of the dots in an array. Symmetrical patterns or patterns that are frequently perceived in the same configuration are faster to enumerate and less error-prone than random arrangements of dots (Mandler and Shebo, 1982; Wender and Rothkegel, 2000; Piazza et al., 2002; Krajcsi et al., 2013). For such “canonical” arrangements of dots, even an extension of the subitizing range was discussed (Mandler and Shebo, 1982). Research indicates that dice patterns hold a special position within canonical arrangements, as they were found to be enumerated even faster than other canonical arrangements (Wender and Rothkegel, 2000). Further, the facilitation effect of symmetrical arrangements and arrangements frequently perceived in the same configuration was reported for the estimation but not for the subitizing range (Mandler and Shebo, 1982; Dehaene and Cohen, 1994).

However, although behavioral findings clearly indicate that the visual quantification process differs for random, canonical, and dice pattern, the question whether the neural response in the IPS during quantification is sensitive to the arrangement of dots is not answered yet. Only few studies investigated the influence of dot pattern arrangement on brain activity during visual quantification (e.g., Piazza et al., 2002). However, studies have been mainly confined to evaluate the influence of dot pattern arrangement on brain activity during subitizing or serial counting. The question whether IPS activity is sensitive to the arrangement of dots during estimation when time restriction prevents serial counting has not yet been addressed systematically.

In the present study, we, therefore, investigated whether set size (subitizing vs. estimation) and spatial arrangement of dots modulate IPS activity during quantification. To address these questions, we conducted an fMRI study in which participants had to enumerate briefly presented dot patterns with random, canonical, and dice arrangement both within the subitizing *and* the estimation range. We pursued a two-step approach to assess processing differences in the IPS between small and large numerosities and between different spatial arrangements: in a first step, we evaluated whether the amplitude of the neuronal response in the IPS was sensitive to set size by means of a region of interest (ROI) analysis. When subitizing and estimation processes differ with respect to their recruitment of critical IPS areas this should be reflected in a distinctive discontinuity in the slopes of IPS activity, when the numerosity of dots in a pattern exceeds four dots (analog to previous behavioral findings). To investigate this transition between subitizing and estimation processes in the IPS we analyzed the linear slopes of IPS activity as a function of numerosity.

In a second step, we evaluated whether the spatial arrangement of dots in an array influenced IPS activity during visual quantification. For this purpose, we followed a similar procedure as Lyons et al. (2015) who investigated whether symbolic and non-symbolic numbers are coded qualitatively differently in the IPS. However, we asked the question whether non-symbolic numbers (i.e., dot patterns) are coded qualitatively different in the IPS, depending on the spatial arrangement of elements in a set (i.e., the spatial arrangement of dots in a pattern). To address this question, we first assessed with a ROI analysis whether the amplitude of IPS activity is influenced by the spatial arrangement of dots in a set during quantification. Behavioral studies reported a facilitation for canonical over random arrangements in the estimation but *not* in the subitizing range (Mandler and Shebo, 1982; Dehaene and Cohen, 1994). Consequently, we expected that IPS activity should be sensitive to the spatial arrangement of dots in the estimation but not in the subitizing range.

Second, we evaluated whether activation patterns in the IPS were influenced by the spatial arrangement of dots. Based on similarity relations between activation patterns in the IPS, revealed by a representational similarity analysis (RSA, Kriegeskorte et al., 2008) it was then possible to infer properties of the underlying neural representation. Thereby, we were able to determine the precision of the magnitude representation for random, canonical, and dice arrangements. Based on behavioral studies indicating a facilitation for arrangements with figural spatial features, we hypothesized that the magnitude representation of canonical and dice arrangements are more precise than that of random arrangements. This should be reflected by the width of respective bell-shaped (tuning) curves indicating similarity between activity patterns in the IPS.

Taken together, based on above theoretical considerations and results of previous imaging studies investigating processes of visual quantification, we derived the following hypothesis:

(1) We expected to observe a signature for a transition between subitizing and estimation processes, reflected by the neural response of IPS, a critical brain area for magnitude processing. In line with previous behavioral findings, which identified a distinctive discontinuity in the slopes of response speed and accuracy during visual quantification whenever an array exceeded four elements, we expected to observe an analog discontinuity in the slope of IPS activation when the numerosity of dots in a visual quantification task exceeds four.
(2) Previous studies reported a facilitation of response times for canonical and dice arrangements as compared to random arrangements of dots during quantification, but only within the estimation range. This indicates that spatial figural features of such structured non-symbolic numerical information impact magnitude processing only in the estimation range. Therefore, we hypothesized weaker IPS response for canonical and dice arrangements than for random arrangements within estimation range.
(3) Finally, we suggested that a more precise neural representation of number magnitude in the IPS for structured stimuli could potentially explain why quantification performance for canonical and dice arrangements is faster and less prone to error than quantification of random arrangements. Consequently, we expected narrower tuning curves for numerosities depicted as dice or canonical arrangement than for those depicted as random arrangement of dots, when comparing IPS activation patterns by means of RSA. This would support the idea of a more precise representation of numerosity in the IPS for structured non-symbolic numerical stimuli with spatial figural features.

## Materials and Methods

### Participants

Twenty-four right-handed volunteers (16 women, mean age = 24 years; *SD* = 6) participated in the study. All subjects gave written informed consent in accordance with the Declaration of Helsinki. The protocol was approved by the Ethics Committee of the Medical Faculty of the University of Tübingen. Participants had normal or corrected to normal vision and reported no previous history of neurological or psychiatric disorders.

### Stimuli and Design

**FIGURE 1:**
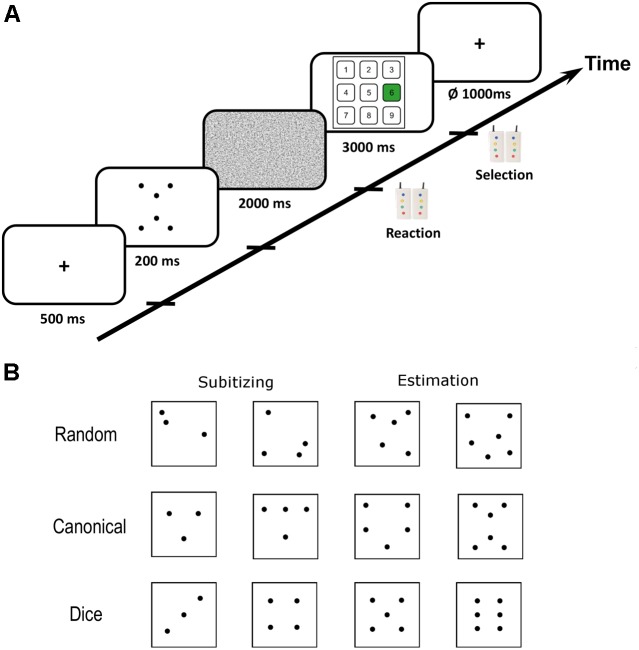
**(A)** Scematic illustration of the trial procedure. Each trial started with a fiaxtion period after which the critical dot pattern was presented for 200 ms. Subsequentially, a noise mask was presented that prevented afterimages. Participants had to respond as soon as they recognized the numerosity of a dot pattern with button press. Subsequently, a visual number pad appeard on the screen and participants had to select the respective number with left and right button presses. **(B)** Representative examples of dot patterns with random, canonical and dice arrangement for subitizing and estimation range. Examples of all dot patterns used in the present study are depicted in **Appendix A**.

Dot pattern arrangements were adapted from Wender and Rothkegel (2000). The number of dots in a pattern ranged from two to eight. Dot pattern stimuli were divided into canonical and random patterns based on the typicality of each dot pattern for the respective numerosity (see Wender and Rothkegel, 2000 for procedure). For each number of dots, three random and three canonical patterns were prepared. Behavioral results indicate that dice patterns occupy a special position within canonical arrangements because dot quantification for these patterns is extremely fast and precise (Mandler and Shebo, 1982; Simons and Langheinrich, 1982; Wender and Rothkegel, 2000). Consequently, we subdivided the canonical patterns for the number range 2–6 into “dice” and “canonical” (=non-dice) arrangements. Therefore, we used three different kinds of dot arrangements in the present study: random (range: 2–8), canonical (range: 2–8), and dice (range: 2–6) patterns. We restricted dice patterns to the range of 2–6, because patterns for this number range are most common on traditional cubic western dice. Representative examples of dot patterns for all three arrangements within subitizing and estimation range are provided in **Figure 1B**. Examples of all dot patterns used in the present study can be found in **Appendix A**.

### Procedure

All participants underwent two functional runs of visual quantification comprising 168 trials each, resulting in 336 critical quantification trials in total. Each experimental trial started with the presentation of a fixation cross (500 ms) followed by the brief presentation of the dot pattern for 200 ms (see **Figure 1A**). Subsequently, a random noise mask was presented for 2000 ms to prevent afterimages. During the presentation of the noise mask, participants had to respond as soon as they recognized the numerosity of the dot pattern by pressing an MRI compatible response button in the right hand. Next, a number pad appeared on the screen for 3000 ms on which participants had to select the numerosity by navigating to the respective number on the number pad with left and right button presses. Each trial was followed by a jittered inter-trial-interval of 1000 ms on average (ranging from 750 to 1250 ms). Initial button presses after presentation of the dot patterns were used to record reaction times (RT) and selected numbers on the number pad entered performance data analysis.

All stimuli were projected on a screen above the participant’s head. Participants viewed the stimuli through a mirror mounted on the head coil of the scanner. Foam pads were used to minimize head movements within the head coil during fMRI acquisition. Total scanning time was approximately 50 min. A baseline (rest) condition was accomplished by including 20% null events in the paradigm.

### Eye Tracking

To ensure that eye movement patterns did not differ between stimulus conditions within and outside the subitizing range as well as all three stimulus arrangements (random, canonical, and dice patterns) we recorded eye movements during all fMRI sessions with an MR compatible tracking device (EyeLink 1000 Plus, SR Research Ltd., Ottawa, ON, Canada). Preprocessing of the eye tracking data included selection of stimulus presentation periods and saccade/fixation detection. Afterward, the distance of gaze from the fixation cross was calculated for every data point. We sorted the data for each subject by arrangement (random, canonical, and dice patterns) and numerosity. We summarized the data per subject by averaging numerosities within (i.e., trials with two to four dots) and outside the subitizing range (i.e., trials with five to eight dots) arrangement condition. Gaze data of the whole stimulus presentations went into later data analysis. Fixation periods, cue events and response periods were discarded from the analysis. Five subjects had to be excluded for the eye tracking analysis due to poor data quality.

### MRI/fMRI Acquisition

A high-resolution T1-weighted anatomical scan was acquired with a 3T Siemens Magnetom Prisma MRI system (Siemens AG; Erlangen, Germany) equipped with a 64–channel head matrix coil (TR = 2300 s, matrix = 256 mm × 256 mm, 176 slices, voxel size = 1.0 mm × 1.0 mm × 1.0 mm; FOV = 256 mm, TE = 2.92 ms; flip angle = 8°). The anatomical scan was performed after the functional runs were completed.

Functional images were obtained using gradient-echo Echo planar imaging (TR = 2400 ms; TE = 30 ms; flip angle = 80°; FOV = 220 mm, 88 × 88 matrix; 42 slices, voxel size = 2.5 mm × 2.5 mm × 3.0 mm, gap = 10%).

FMRI data were analyzed with SPM12^1^. Images were motion corrected and realigned to the mean functional image of each participant. Imaging data were then normalized into standard stereotaxic MNI space (Montreal Neurological Institute, McGill University, Montreal, QC, Canada). Images were resampled every 2.5 mm using 4th degree spline interpolation and smoothed with a 5 mm FWHM Gaussian kernel to accommodate inter-subject variation in brain anatomy and to increase signal-to-noise ratio in the data. The data were high-pass filtered (128 s) to remove low-frequency noise components and corrected for autocorrelation assuming an AR(1) process. Brain activity was convolved over the experimental trials with the canonical haemodynamic response function (HRF) and its first time derivative. The resulting design matrices comprised 19 experimental regressors, one for each combination of numerosity and arrangement. To capture residual movement-related artifacts, we included six motion regressors of no interest. Individual participants’ contrast images obtained from first-level analysis entered second-level analysis. For the second level analysis we contrasted estimated beta weights for the respective conditions using the flexible factorial design option within SPM12.

### Region of Interest Analysis

We defined two anatomical regions of interest (ROIs) using the SPM Anatomy toolbox v2.0 (Eickhoff et al., 2005, 2006, 2007): an anatomical ROI covering (1) left intraparietal sulcus (hIP1, hIP2, and hIP3) and (2) right intraparietal sulcus (hIP1, hIP2, and hIP3), respectively, because differential contributions of left and right IPS to the processing of numerical magnitude have been discussed (Chochon et al., 1999; Ansari, 2007; Cohen Kadosh et al., 2007; Piazza et al., 2007). We used the SPM toolbox MarsBar^2^ for ROI definition and the later ROI analysis.

### Behavioural Analysis

We analyzed both error rates (ERs) and RTs of correct responses. Five participants were excluded from analysis: three could not be analyzed due to technical problems, and two participants committed more than 20% errors in the subitizing range. ERs were analyzed using a generalized linear mixed effects model (GLME) with a binomial error distribution and logit as link function utilizing the R package lme4 (Bates et al., 2015). Fixed effects in this model were the categorical predictors “arrangement” (i.e., random, canonical, vs. dice pattern) and “number range” (i.e., subitizing vs. estimation) as well as their interactions. We did not include the continuous predictor “numerosity” (i.e., number of presented dots), because ERs were zero for most of the participants in the subitizing range of the dice condition and, thus, the slope could not be estimated. Both predictors (i.e., “arrangement” and “range”) were effect coded. Moreover, we included a random intercept for participants in the GLME.

For analysis of RTs, linear mixed effect models (LME) were applied. Prior to the analysis of RTs, a trimming procedure was conducted excluding all RTs smaller than 200 ms. As the distribution of RTs was right-skewed, we applied a log-transformation (Ratcliff, 1993). In a next step, we ran a LME with log-transformed RTs (log RT) as dependent variable, “arrangement,” “number range,” and “numerosity” as fixed effects as well as their interactions, and the maximum random effects structure (i.e., including all fixed effects as random effects as well as a random intercept for participants; Barr et al., 2013). The continuous predictor variable “numerosity” was centered separately for all conditions of stimulus arrangement and range. Then, we applied a model-based trimming procedure by *z*-standardizing residuals of the LME and excluding all log RTs with residuals deviating more than ±3 *SD* (Baayen and Milin, 2015). In total (including eliminating erroneous responses), this reduced the data set by less than 10%.

Both for the GLME and the LME *p*-values were calculated using likelihood ratio tests (LRT) and the R package afex (Singmann et al., 2016). Furthermore, we conducted *post hoc* analyses using the R package multcomp (Hothorn et al., 2008). In order to account for multiple testing, we adjusted the *p*-values employing the Benjamini–Hochberg procedure (Benjamini and Hochberg, 1995). Plots were drawn using the R packages ggplot2 (Wickham, 2009) and cowplot (Wilke, 2016).

## Results

### Behavioral Data

**FIGURE 2:**
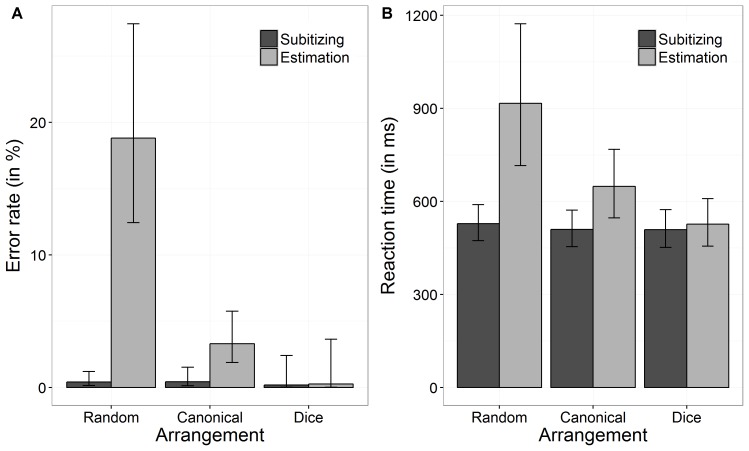
Back transformed error rates (ER) **(A)** and reaction times **(B)** as a function of arrangement and number range. Error bars indicate 95% confidence intervals.

An overview of performance data (i.e., ERs and RTs) separately for the three conditions of arrangement (random, canonical, and dice) as well as for the two number ranges (subitizing and estimation) is given in **Figure 2**.

#### Error Rates

We investigated the effect of arrangement and number range on task performance (i.e., errors made in the numerosity estimation task) using a GLME. The results are summarized in **Table 1**. Our results revealed a significant interaction between “arrangement” and “number range,” which is depicted in **Figure 2A**. To analyze this interaction, we first tested whether ERs differed between arrangement conditions by running two GLME for subitizing and estimation range, separately. For the subitizing range, there was no significant effect of arrangement on ERs, χ^2^(2) = 0.94, *p* = 0.625. Participants’ ERs in the subitizing range for random patterns were: log odds = -5.51, *SE* = 0.42, back transformed ER = 0.40%, for canonical patterns: log odds = -5.44, *SE* = 0.49, back transformed ER = 0.43%, and for dice patterns: log odds = -6.35, *SE* = 1.02, back transformed ER = 0.17%. Thus, the ERs did not differ significantly between the three arrangement conditions. In contrast, for the estimation range a significant effect of arrangement on ERs was observed, χ^2^(2) = 298.78, *p* < 0.001. Pairwise comparisons revealed a significant difference between ERs in the random and the canonical condition (*z* = 12.94, *p* < 0.001), between the random and the dice condition (*z* = 4.45, *p* < 0.001), as well as between the canonical and the dice condition (*z* = 2.53, *p* = 0.012). In the estimation range, participants performed worse in the random arrangement condition (log odds = -1.46, *SE* = 0.19, back transformed ER = 18.81%) than in the canonical arrangement (log odds = -3.38, *SE* = 0.22, back transformed ER = 3.30%) and the dice condition (log odds = -5.94, *SE* = 1.02, back transformed ER = 0.26%). Thus, the main effect of “arrangement” should not be interpreted, as there was no coherent effect of arrangement on task performance.

**Table 1:** Results of the (generalized) linear mixed effects model for errors, log-transformed RT (log RT) and percent signal change (PSC).

Dependent variable	Fixed effect	df	χ^2^	*p*-value
Error	Range	1	9.92	0.002
	Arrangement	2	25.49	<0.001
	Range × Arrangement	2	12.44	0.002
Log RT	Numerosity	1	12.04	<0.001
	Range	1	23.86	<0.001
	Arrangement	2	23.76	<0.001
	Numerosity × Range	1	1.77	0.183
	Numerosity × Arrangement	2	17.81	<0.001
	Range × Arrangement	2	25.65	<0.001
	Numerosity × Range × Arrangement	2	9.75	0.008
PSC	Numerosity	1	7.17	0.007
	Range	1	23.18	<0.001
	Arrangement	2	7.55	0.023
	IPS	1	16.79	<0.001
	Numerosity × Range	1	7.71	0.006
	Numerosity × Arrangement	2	18.44	<0.001
	Numerosity × Hemisphere	1	0.10	0.752
	Range × Arrangement	2	12.01	0.002
	Range × Hemisphere	1	0.11	0.739
	Arrangement × Hemisphere	2	1.60	0.449
	Numerosity × Range × Arrangement	2	34.74	<0.001
	Numerosity × Range × Hemisphere	1	0.50	0.480
	Numerosity × Arrangement × Hemisphere	2	0.03	0.985
	Range × Arrangement × Hemisphere	2	0.08	0.962
	Numerosity × Arrangement × Range × Hemisphere	2	0.18	0.914


Secondly, we tested, whether performance between subitizing and estimation range differed for each arrangement condition, separately. ERs of the random and the canonical condition differed significantly between subitizing and estimation range (random: *z* = -10.45, *p* < 0.001, canonical: *z* = -4.38, *p* < 0.001). However, there was no significant difference regarding ERs between subitizing and estimation range for the dice condition (*z* = -0.29, *p* = 0.773). Therefore, also the main effect of range should not be interpreted as there was no consistent pattern of range.

#### Reaction Times

Analog to the analysis of ERs, we investigated the effect of arrangement and number range on log-transformed RT. The respective results of the LME are given in **Table 1**.

In line with the results for ERs, we observed a significiant interaction between arrangement and number range (see **Figure 2B**). Again, to analyze this interaction, we first ran two LMEs with arrangement as fixed effect for subitizing and estimation range, separately. The effect of arrangement on log RTs was significant for subitizing, χ^2^(2) = 9.20, *p* = 0.010, as well as for estimation range, χ^2^(2) = 26.22, *p* < 0.001. For the subitizing range, participants’ log RT for the random, the canonical, and the dice condition were 6.27 ms (*SE* = 0.05 ms, back transformed RT = 528.29 ms), 6.23 ms (*SE* = 0.05 ms, back transformed RT = 509.59 ms), and 6.23 ms (*SE* = 0.05 ms, back transformed RT = 508.79 ms). Pairwise comparisons indicated that only log RT of random and canonical conditions differed significantly (*z* = -2.42, *p* = 0.046), whereas other comparisons were not significant (dice vs. canonical: *z* = -0.08, *p* = 0.940; dice vs. random: *z* = 1.92, *p* = 0.082). For the estimation range, participants’ log RT for the random, the canonical, and the dice condition were 6.82 ms (*SE* = 0.11 ms, back transformed RT = 916.22 ms), 6.47 ms (*SE* = 0.07 ms, back transformed RT = 648.02 ms), and 6.27 ms (*SE* = 0.06 ms, back transformed RT = 526.85 ms). Pairwise comparisons revealed that all three comparisons were significant (dice vs. canonical: *z* = -6.23, *p* < 0.001; dice vs. random: *z* = -7.57, *p* < 0.001; canonical vs. random: *z* = -6.77 *p* < 0.001).

Regarding differences between subitizing and estimation range, our results revealed that log RT differed significantly between the subitizing and the estimation range in the random arrangement condition as well as in the canonical arrangement condition (random: *z* = -7.72, *p* < 0.001; canonical: *z* = -6.93, *p* < 0.001). However, in the dice condition no significant difference between subitizing and estimation range was found (*z* = -1.26, *p* = 0.209).

**FIGURE 3:**
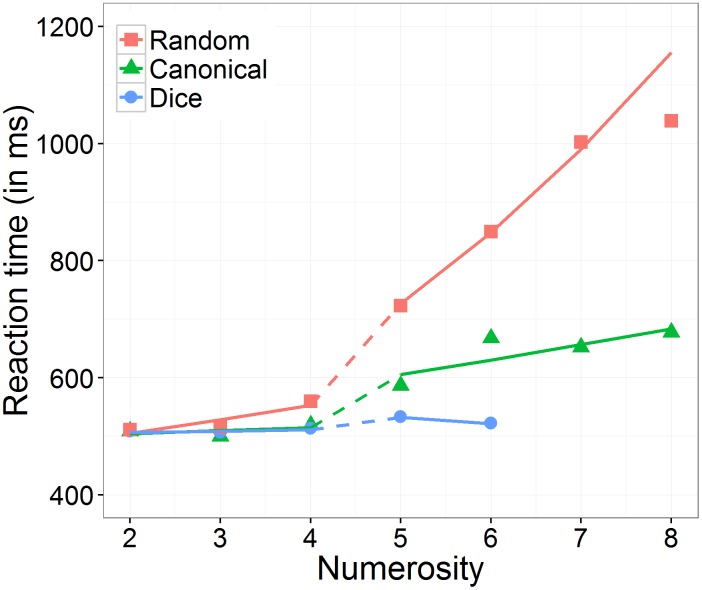
Effect of numerosity on predicted (including random effects) back transformed reaction times, separately for the number range conditions (subitizing and estimation) and the arrangement conditions (random, canonical, and dice).

Moreover, we found a significant three-way interaction between numerosity, range, and arrangement, which is depicted in **Figure 3**. First, we investigated whether the effect of numerosity differed significantly from zero in the subitizing as well as in the estimation range separately for each arrangement condition. The effect of numerosity on log RT was significant in subitizing as well as in estimation range for the random condition (subitizing: 0.045 ms, *SE* = 0.017 ms, *z* = 2.89, *p* = 0.008; estimation: 0.155 ms, *SE* = 0.021 ms, *z* = 7.46, *p* < 0.001) and in the estimation range for the canonical condition (0.010 ms, *SE* = 0.015 ms, *z* = 4.06, *p* < 0.001), whereas it was not significant in subitizing range for the canonical condition (0.041 ms, *SE* = 0.010 ms, *z* = 0.69, *p* = 0.717) and in both ranges for the dice condition (subitizing: 0.004 ms, *SE* = 0.019 ms, *z* = 0.22, *p* = 0.826; estimation: -0.021 ms, *SE* = 0.039 ms, *z* = -0.53, *p* = 0.717).

Second, we analyzed the three-way interaction. To do so, we evaluated the effect of arrangement on the slope of numerosity, separately for the two number ranges subitizing and estimation. In the subitizing range, there was no significant interaction between numerosity and arrangement, χ^2^(2) = 4.82, *p* = 0.090. In contrast, in the estimation range, we observed a significant interaction between numerosity and arrangement, χ^2^(2) = 20.82, *p* < 0.001. *Post hoc* comparisons indicated that the estimated slope (i.e., effect of numerosity on log RT) was significantly larger in the random than in the canonical condition (*z* = 5.71, *p* < 0.001), as well as in the dice condition (*z* = 3.95, *p* < 0.001). However, the estimated slope in the canonical condition was not significantly larger than in the dice condition (*z* = 1.52, *p* = 0.128).

Next, we analyzed the effect of number range on the slope of numerosity, separately for the three arrangement conditions. We observed that in the dice condition as well as in the canonical condition, the effect of numerosity on log RT did not differ significantly between the subitizing range and the estimation range (dice: *z* = 0.58, *p* = 0.564; canonical: *z* = -1.77, *p* = 0.115), while in the random condition the effect of numerosity on log RT was significantly larger in the estimation range than in the subitizing range (random: *z* = 5.23, *p* < 0.001).

#### Eye Tracking

For each subject and stimulus condition, we calculated the mean gaze position as well as the standard deviation of gaze position as a measure of eye movement distribution. We conducted two separate 2 × 3 ANOVAs with the factors number range (subitizing vs. estimation) and arrangement (random vs. canonical vs. dice) with the dependent variables mean gaze position and standard deviation of gaze position, respectively. For both dependent variables we neither observed a significant main effect: mean gaze position: range: *F*(1,18) = 0.27, *p* = 0.61, arrangement: *F*(2,17) = 1.66, *p* = 0.22; standard deviation: range: *F*(1,18) = 0.55, *p* = 0.47, arrangement: *F*(2,17) = 2.60, *p* = 0.11) nor an interaction [average gaze position: *F*(2,17) = 1.33, *p* = 0.29; standard deviation: *F*(2,17) = 0.05, *p* = 0.99].

### Imaging Data

#### Whole Brain Analysis

Prior to the ROI analyses, we will present contrasts for the three different spatial arrangements (i.e., dice, canonical, and random) separately for subitizing and estimation range to provide the interested reader with an overview of brain activation patterns elicited by the respective conditions.

##### Subitizing range

The comparison of brain activation for different spatial arrangements within the subitizing range revealed no suprathreshold clusters of activation.

##### Estimation range

###### Dice vs. random

**FIGURE 4:**
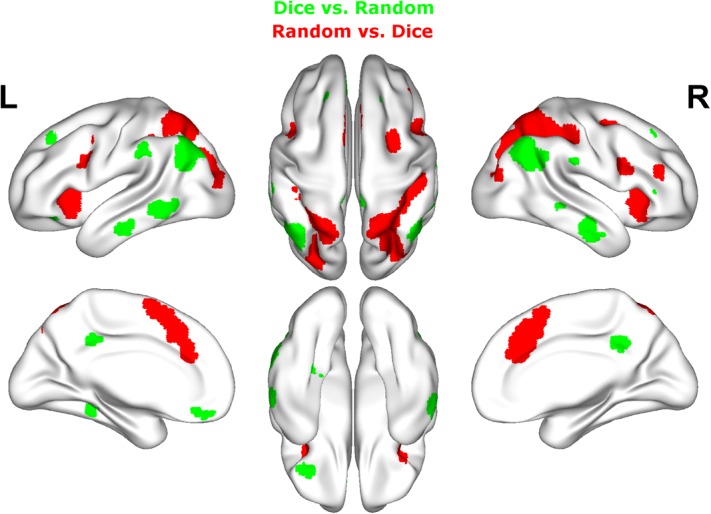
Results of the whole brain analysis. The contrast between dice and random arrangements within the estimation range (depicted in green) revealed activity in bilateral angular gyrus, supramarginal gyrus, middle temporal gyrus, inferior frontal gyrus as well as left hemispheric activation of fusiform gyrus. The contrast between radnom and dice arrangements within estimation ranged (depicted in red) revealed stronger activation in bilateral IPS, anterior and middle cingulate cortex (ACC, MCC), middle frontal gyrus as well as inferior frontal gyrus. Activation is depicted on a 3D rendered surface (all at *P*_cluster-corr_ < 0.001, cluster size of *k* = 15 voxels).

Contrasting dice with random arrangements in the estimation range revealed bilateral activation in angular gyrus (PGa), supramarginal gyrus (PF) as well as middle temporal, inferior frontal and superior frontal gyrus. Left hemispheric clusters were observed in the frontal pole, the inferior temporal gyrus as well as in the fusiform gyrus and subiculum (extending into CA1 of the hippocampus). Right hemispheric activation comprised a cluster in the retrosplenial cortex (see **Figure 4** depicted in green, **Table 2**).

**Table 2:** Results of the whole brain analysis for different spatial arrangements within estimation range.

Contrast	Brain region	MNI (x, y, z)			Cluster size	*T*
**Dice vs. random**						
	RH angular gyrus (PGa) (extending into superior temporal gyrus)	50	-71	43	500	7.70
	LH angular gyrus (PGa)	-51	-76	38	402	6.18
	LH supramarginal gyrus (PF)	-66	-39	40	51	5.34
	RH supramarginal gyrus (PF)	65	-31	30	45	4.27
	RH retrosplenial cortex	12	-51	40	97	5.50
	RH middle temporal gyrus	60	-19	-18	92	5.39
	LH middle temporal gyrus	-58	-24	-18	28	5.04
	LH middle temporal gyrus	-63	-49	-3	97	5.84
	LH inferior temporal gyrus^∗^	-58	-61	0		4.52
	LH fusiform gyrus	-31	-44	-10	104	4.27
	LH subiculum^∗^	-26	-31	-18		4.01
	LH inferior frontal gyrus (p. orbitalis)	-33	32	-8	22	4.83
	RH inferior frontal gyrus (p. triangularis)	55	35	10	33	4.59
	RH superior frontal gyrus	25	32	58	20	4.67
	LH superior frontal gyrus	-23	32	55	59	4.25
	LH area Fp2	-1	47	-5	56	5.02
**Random vs. dice**						
	RH intraparietal sulcus (hIP1, hIP2, hIP3)	25	-64	65	783	9.53
	LH intraparietal sulcus (hIP2, hIP3)	-23	-64	63	335	8.11
	RH middle cingulate cortex (extending into anterior cingulate cortex)	7	24	38	604	7.31
	LH inferior frontal gyrus (p. opercularis)	-46	7	33	32	5.60
	RH inferior frontal gyrus (p. opercularis)	50	7	30	53	5.88
	RH middle frontal gyrus	40	34	25	97	5.51
	LH middle frontal gyrus	-23	2	55	63	4.47
	RH thalamus	2	-34	3	4.75	4.75
	RH caudate nucleus	15	12	8	174	6.06
	RH insula lobe	32	22	3	250	9.13
	LH insula lobe	-31	22	8	229	8.88
	LH putamen^∗^	-23	4	-5		4.83
	LH precentral gyrus	-11	-76	18	70	4.44
	LH calcarine gyrus	-11	-76	18	70	4.44
	LH middle occipital gyrus	-28	-91	28	240	6.94
	LH cerebellum	-28	-71	-23	144	7.33
**Canonical vs. random**						
	LH angular gyrus (PGa)	-43	-69	50	437	5.28
	RH angular gyrus (PGp)	50	-74	43	271	6.44
	RH supramarginal gyrus (PF)	60	-29	33	33	4.01
	LH middle temporal gyrus	-61	-51	-5	199	4.29
	LH inferior temporal gyrus^∗^	-58	-21	-23		3.87
	RH inferior temporal gyrus	60	-19	20	143	4.98
	RH fusiform gyrus	32	-41	-10	267	5.61
	LH fusiform gyrus	-31	-44	-10	196	4.82
	LH area Fp2	-6	42	-13	289	5.18
	LH inferior frontal gyrus (p.orbitalis)	-36	29	-13	69	5.20
	RH inferior frontal gyrus (p.orbitalis)	27	29	-13	50	5.18
**Random vs. canonical**						
	RH intraparietal sulcus (hIP1, hIP3)	7	23	-63	50	4.83
	RH middle cingulate cortex extending into anterior cingulate cortex	5	29	35	976	9.75
	RH anterior cingulate cortex^∗^	4	34	31		7.93
	RH pre-supplementary motor area (preSMA)^∗^	10	14	55		8.53
	RH inferior frontal gyrus	-3	22	45	443	7.93
	RH insular lobe^∗^	40	17	-5		8.60
	RH middle frontal gyrus	40	34	28	137	6.78
	LH putamen	-15	9	5	52	6.03


*^∗^Secondary peak. MNI, Montreal Neurological Institute; T, t-value; P_cluster-corr_ < 0.001 (k = 15 voxels).*

###### Random vs. dice

The contrast of random and dice arrangements within the estimation range revealed bilateral IPS activation (hIP1, hIP2, and hIP3) extending into the superior and middle occipital gyrus. Further bilateral clusters were observed in the inferior frontal (p. opercularis) and middle frontal gyrus as well as in the insular lobe. Left hemispheric activation was found in the putamen, the precentral gyrus and the calcarine gyrus. Finally, right hemispheric clusters were observed in middle cingulate cortex (extending into anterior cingulate cortex), thalamus and caudate nucleus (see **Figure 4** depicted in red, **Table 2**).

###### Canonical vs. random

**FIGURE 5:**
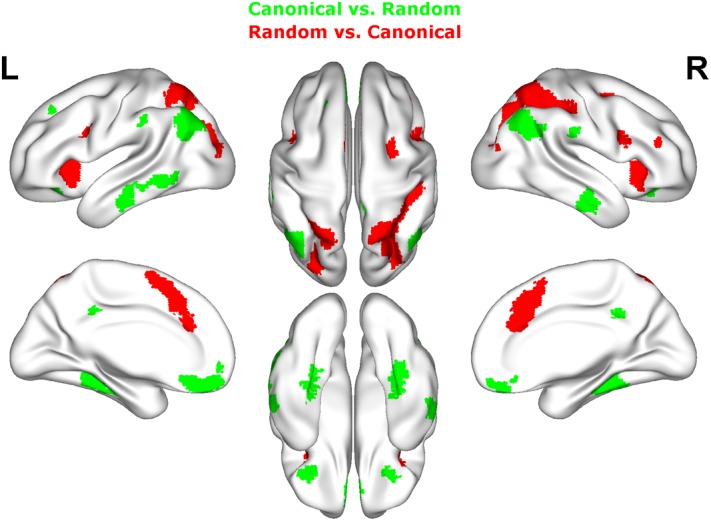
Results of the whole brain analysis. The contrast between canonical and random arrangements within the estimation range (depicted in green) revealed activity in bilateral angular gyrus, inferior temporal gyrus as well as fusiform gyrus and inferior frontal gyrus. The contrast between radnom and canonical arrangements within estimation ranged (depicted in red) revealed stronger activation in bilateral IPS, anterior and middle cingulate cortex (ACC, MCC), middle frontal gyrus as well as inferior frontal gyrus and pre-supplemenatry motor areas (pre-SMA). Activation is depicted on a 3D rendered surface (all at *P*_cluster-corr_ < 0.001, cluster size of *k* = 15 voxels).

Contrasting brain activation for canonical and random arrangements within the estimation range revealed bilateral activation of the angular gyrus (PGa, PGp), the inferior temporal gyrus as well as the fusiform gyrus (extending into CA1 of the hippocampus). Further a bilateral cluster was observed in the inferior frontal gyrus (p.orbitalis). Left hemispheric activation comprised clusters in the middle temporal gyrus and the frontal pole region. Finally, right hemispheric activation was observed in the supramarginal gyrus (see **Figure 5** depicted in green, **Table 2**).

###### Random vs. canonical

Contrasting brain activation for random and canonical arrangements within the estimation range revealed right hemispheric activation of the intraparietal sulcus (hIP1, hIP3) the middle cingulate cortex and the anterior cingulate cortex (see **Figure 5** depicted in red, **Table 2**).

For sake of completeness we provide the remaining contrasts between arrangements within the estimation range as well as simple contrasts against baseline in the **Appendix B**.

#### Region of Interest Analysis

The mean percent signal changes (PSC) relative to fixation within left and right IPS ROI were extracted for each participant and condition using MarsBar toolbox^3^. For the analysis of PSCs linear mixed effect models were used with “arrangement,” “number range,” “numerosity,” and “hemisphere” and their interactions as fixed effects and “number range,” “numerosity,” and “hemisphere” as well as their interactions as random effects. *P*-values were calculated running LRTs using the R package afex (Singmann et al., 2016). In order to account for multiple testing, *p*-values of subsequent *post hoc* tests were adjusted following the Benjamini–Hochberg procedure (Benjamini and Hochberg, 1995).

#### Percent Signal Change Results

An overview of PSC for the conditions of stimulus arrangement (random, canonical, and dice) as well as for the two number ranges (subitizing and estimation) and hemisphere (left and right) is given in **Table 3**. The results of the LME are given in **Table 1**.

**Table 3:** Mean (M), standard deviation (SD), minimum and maximum percent signal change relative to fixation (PSC) for the six conditions of the numerosity estimation task.

			PSC			
			
Arrangement	Number range	Hemisphere	*M*	*SD*	Minimum	Maximum
Random	Subitizing	Left	0.24	0.15	-0.25	0.66
		Right	0.14	0.17	-0.24	0.58
	Estimation	Left	0.42	0.17	-0.04	0.81
		Right	0.33	0.23	-0.04	1.00
Canonical	Subitizing	Left	0.23	0.16	-0.19	0.52
		Right	0.12	0.18	-0.22	0.46
	Estimation	Left	0.38	0.17	-0.24	0.74
		Right	0.28	0.22	-0.22	0.85
Dice	Subitizing	Left	0.28	0.25	-0.37	1.23
		Right	0.16	0.24	-0.37	0.74
	Estimation	Left	0.32	0.23	-0.35	0.82
		Right	0.19	0.28	-0.39	0.85


**FIGURE 6:**
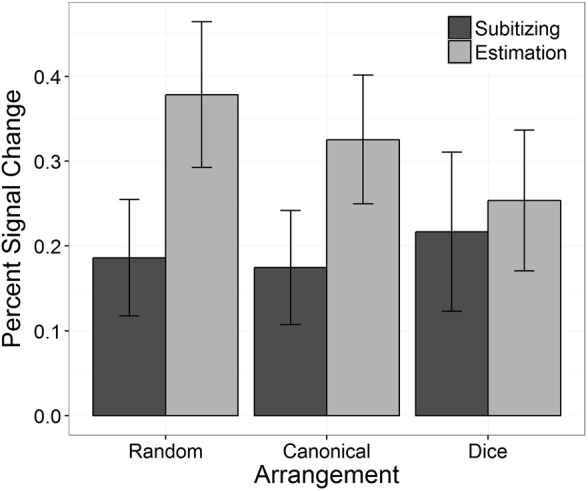
Percent signal change in bilateral IPS as a function of arrangement and number range. Error bars indicate 95% confidence intervals.

The analysis of the PSC revealed a significant interaction between “arrangement” and “number range,” which is depicted in **Figure 6**. To break down this interaction, we ran *post hoc* tests comparing the PSC between the three arrangements separately for subitizing and estimation range. For subitizing range, PSC did not differ significantly between arrangements, χ^2^(2) = 0.95, *p* = 0.623. In contrast, for estimation range a significant effect of arrangement on PSC was observed, χ^2^(2) = 12.10, *p* = 0.002. Pairwise comparisons revealed significant differences in PSC between the random and the canonical condition (*z* = 3.25, *p* = 0.001), between the random and the dice condition (*z* = 3.81, *p* < 0.001), as well as between the canonical and the dice condition (*z* = 2.13, *p* = 0.033).

Our results also revealed that PSC differed significantly between subitizing and estimation range in the random arrangement condition as well as in the canonical arrangement condition (random: *z* = -8.45, *p* < 0.001; canonical: *z* = -5.33, *p* < 0.001). However, in the dice condition no significant difference between the subitizing and the estimation range was detected (*z* = -0.97, *p* = 0.331).

**FIGURE 7:**
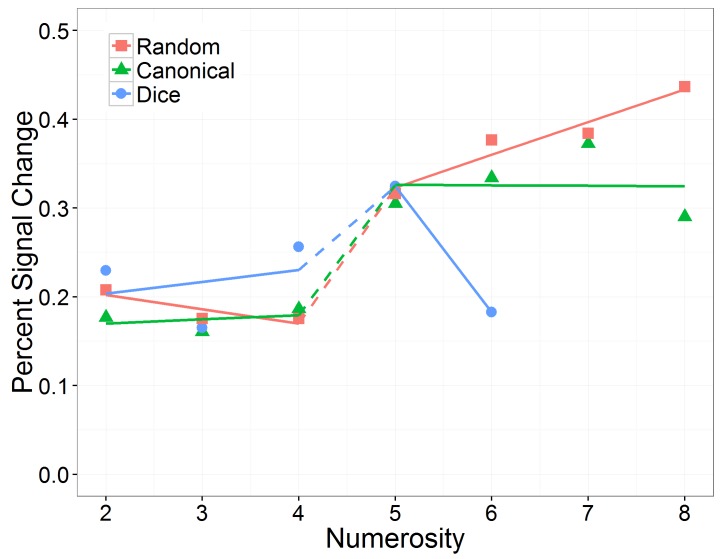
Effect of numerosity on percent signal change, separately for the number range conditions (subitizing and estimation) and the arrangement conditions (random, canonical, and dice).

We further found a significant three-way interaction between numerosity, number range, and arrangement, which is depicted in **Figure 7**. To break down this interaction, we evaluated the influences of the continious predictor “numerosity” and the predictor “arrangement” separately for the subitizing and the estimation range. In the subitizing range, there was no significant interaction between numerosity and arrangement, χ^2^(2) = 2.44, *p* = 0.296. In the estimation range, we observed a significant interaction between numerosity and arrangement, χ^2^(2) = 35.99, *p* < 0.001. We estimated slopes separatly for each arrangement in subitizing and estimation range: In the subitizing range, slope estimates for the random (slope: -0.02; *SE* = 0.01; *z* = -1.16, *p* = 0.490), the canonical (slope: 0.00; *SE* = 0.01; *z* = 0.34, *p* = 0.880) and the dice condition (slope: 0.01; *SE* = 0.01; *z* = 0.96, *p* = 0.506) did not significantly differ from zero. In the estimation range slopes of random (slope: 0.04; *SE* = 0.01; *z* = 4.20, <0.001) and dice (slope: -0.14, *SE* = 0.03; *z* = -5.12, *p* < 0.001) arrangements differed significantly from zero whereas the slope of canonical arrangements did not (slope: 0.00; *SE* = 0.01; *z* = -0.08, *p* = 0.940).

To further investigate the influence of the factor “arrangement” on IPS response during estimation, we compared slope estimates for different arrangements within the estimation range. These pairwise comparisons revealed that slopes differed significantly between random and canonical (*z* = -3.25, *p* = 0.002), random and dice (*z* = -3.81, *p* < 0.001) as well as between canonical and dice arrangements (*z* = 2.13, *p* = 0.033) in the estimation range (**Figure 7**).

Subsequently, we analyzed the effect of number range on the slope of numerosity, separately for the three arrangement conditions. We observed that in the canonical condition, the effect of numerosity on PSC in the IPS did not differ significantly between the subitizing range and the estimation range (*z* = 0.33, *p* = 0.743), whereas in the random condition the effect of numerosity on PSC in the IPS was significantly larger in the estimation range than in the subitizing range (*z* = 5.01, *p* < 0.001). In contrast, in the dice condition the effect of numerosity on PSC in the IPS was significantly smaller in the estimation range than in the subitizing range (*z* = -3.22, *p* = 0.002).

Finally, the main effect of hemisphere was significant (see **Table 3**). It indicated that PSC was larger in the left than in the right IPS (left = 0.31, *SE* = 0.02, *z* = 13.38, *p* < 0.001; right = 0.20, *SE* = 0.03, *z* = 6.70, *p* < 0.001).

#### Representational Similarity Analysis

**FIGURE 8:**
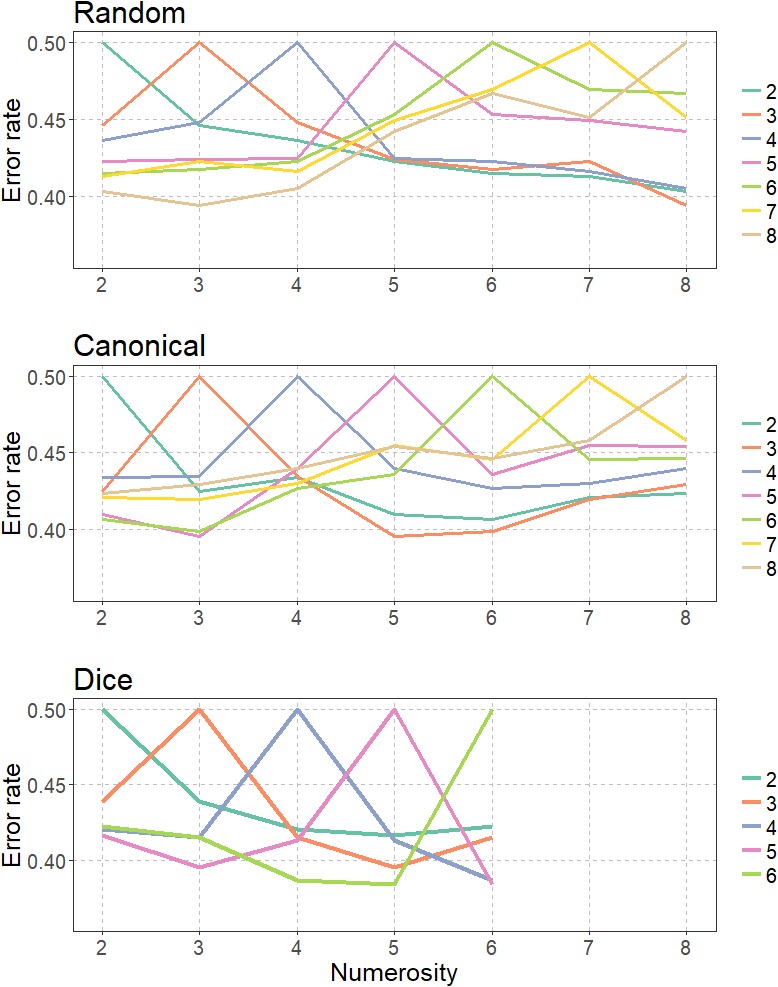
Similarity curves of IPS activity patterns for different numerosities and arrangements.

We further investigated the degree of similarity in IPS activity patterns for different numerosities and arrangements. Therefore, we conducted a logistic regression analysis for each combination of numerosity and arrangement (e.g., can2 vs. dice 5) and determined the classification accuracy for each of the resulting combinations for each participant. We focused this analysis on the anatomically defined bilateral IPS using the SPM Anatomy Toolbox (Eickhoff et al., 2005), because this area is commonly associated with the processing of absolute and relative number magnitude information (e.g., Piazza et al., 2007; Jacob and Nieder, 2009; Arsalidou and Taylor, 2011). The logistic regression analysis was based on β estimates obtained by rerunning the GLM with unsmoothed images. Then, we applied the anatomically defined bilateral IPS mask for each participant and each notation format. In a next step classification accuracies were averaged across participants and transformed into classification ERs. The resulting classification ERs for the different arrangements are depicted in the form of representational similarity curves in **Figure 8** with higher ERs indicating more similar activation patterns resulting in a worse classification performance.

Similarity curves allow for the assessment of the similarity of neural patterns in the IPS for different numbers and arrangements. Based on similarity relations between pairs of numbers it is then possible to infer properties of the underlying neural representation (Kriegeskorte et al., 2008; Lyons et al., 2015).

Highly overlapping similarity curves thereby indicate that activity patterns and thus also the neural representations of those two numbers are highly similar. Contrarily, similarity curves with little to no overlap indicate that the neural representations of two numbers are highly dissimilar.

Based on these similarity values, we estimated the width of the bell-shape similarity curves between numerosities for subitizing and estimation as well as different arrangements using the linear model for numerosity judgments with *w* indicating the width of the Gaussian tuning curves (Pica et al., 2004).

$$f(x)=\frac{1}{\sqrt{2\pi wn}}e^{-\frac{{\left(x-n\right)}^{2}}{{2(wn)}^{2}}}$$

Fitting the linear model using the nls function of R with start value of 0.4 revealed that the width of the bell-shape similarity curves differed between arrangements. Overall *w* was smallest for the dice condition with *w* = 0.094 indicating that IPS activity patterns was most different for the dice condition. *w* of the canonical condition with *w* = 0.241 was in between the *w* of the dice and the random condition. Finally, *w* was largest for the random condition with *w* = 0.276 that IPS activity patterns were most similar in the random condition.

## Discussion

Quantification of visual objects is one of the most basic numerical competencies, present already in infants and observed for various non-human animals. Although this skill seems to route in an evolutionary ancient cognitive system (Brannon, 2006), our knowledge of the neural mechanisms underlying quantification is still rather patchy. Psychophysical studies claimed a qualitative distinction between subitizing – the rapid and accurate perception of small sets – and estimation – an effortless but error-prone and approximate quantification process (Kaufman et al., 1949; Mandler and Shebo, 1982; Trick and Pylyshyn, 1994; Wender and Rothkegel, 2000). Moreover, it was shown that quantification is faster and more precise for symmetrical dot patterns and patterns that are frequently perceived in the same configuration (e.g., dice patterns; Mandler and Shebo, 1982; Wender and Rothkegel, 2000). However, the question how activity of the IPS – the key area involved in quantification of small and large visual arrays – is modulated by the set size of a visual array and whether IPS activity is sensitive to the spatial arrangement of elements is not fully answered yet. The current study addressed these questions by evaluating the neuronal response in bilateral IPS, a key area for number magnitude processing, during visual quantification of random, canonical, and dice dot patterns within the subitizing and the estimation range.

Our behavioral data clearly reflected a qualitative change between quantification of small (subitizing range) and large visual arrays (estimation range). We observed a characteristic discontinuity in the slopes of response times and ERs between the subitizing and the estimation range. The increase in response times and ERs was negligible within the subitizing range but substantial for arrays exceeding four elements. Therefore, RT and ER patterns reflected the transition between a subitizing and estimation process observed in various other studies (e.g., Revkin et al., 2008). Consequentially, the implemented quantification paradigm with non-verbal responses revealed similar findings to previous studies that used a verbal response outside of the MR scanner (e.g., Mandler and Shebo, 1982; Wender and Rothkegel, 2000).

Moreover, behavioral data revealed that quantification performance was also influenced by the spatial arrangement of dots in an array (see also Mandler and Shebo, 1982; Wender and Rothkegel, 2000; Piazza et al., 2002; Krajcsi et al., 2013 for similar findings). This influence of arrangement on quantification depended on the set size. A facilitation of response times when quantifying canonical and dice arrangements over random arrangements was observed in the estimation but not in the subitizing range: the increase in response times and ERs in the estimation range was steeper for random dot patterns than for canonical and dice dot patterns. For dice patterns, we observed no increase in RTs and ERs for increasing numerosity, suggesting a larger subitizing range for these highly overlearned arrangements. In sum, our behavioral findings are in agreement with previous studies (e.g., Mandler and Shebo, 1982; Dehaene and Cohen, 1994; Wender and Rothkegel, 2000; Piazza et al., 2002) supporting the validity of our MRI compatible non-verbal version of the subitizing task.

Before we outline in detail how activity of IPS, the key area for magnitude processing, is modulated by set size and spatial arrangement of elements in a given set, we will briefly discuss fronto-parietal networks responsive to set size and arrangement as identified by the whole brain analysis.

### The Influence of Arrangement on Visual Quantification

On whole brain level, contrasting activation for different arrangements within the subitizing range revealed no suprathreshold voxels of activity. This mirrors the behavioral data that showed no facilitating effect of canonical and/or dice over random arrangements for subitizing trials. This can be explained by inherent spatial figural properties of random arrangements within subitizing range. For instance, three randomly distributed dots can often be perceived as a triangle and four random dots as a rectangle, trapeze or another specific spatial figure. Consequently, such inherent spatial features may have reduced differences between the types of arrangements in the subitizing range, so that we were not able to detect differences in brain activation for different spatial arrangements on a whole brain level.

Within estimation range, visual quantification of non-structured (random) and structured (canonical and dice) dot patterns revealed two fundamentally distinct patterns of activation. Stimuli with spatial figural features (i.e., dice and canonical patterns) elicited stronger activation in a distributed network of areas associated with familiarity processing comprising, amongst others, bilateral angular gryus, supramarginal gyrus, and retrosplenial cortex (cf. Shah et al., 2001; Sugiura et al., 2005; Horn et al., 2016). Interestingly, this pattern of activation broadly replicates findings of studies that compared brain activation for symbolic and non-symbolic numerical input (e.g., Holloway et al., 2010). In line with that, recruitment of middle temporal and inferior frontal brain regions, associated with semantic processing (Röder et al., 2002; Chou et al., 2009; Visser et al., 2012) indicate that structured and overlearned non-symbolic numerical stimuli like dice pattern might gain iconic properties and thus carry semantic content beyond their numerical magnitude. Furthermore, structured arrangements with such spatial figural features elicited activation in fusiform gyrus. This area of the ventral visual stream plays a central role in differentiating between different categories of objects and is particularly responsive to over-trained visual stimuli (e.g., Rhodes et al., 2004; Tyler et al., 2013; Zhang et al., 2016). Therefore, involvement of the fusiform gyrus further suggested a substantially different processing of highly structured non-symbolic numerical information in the brain.

In contrast, processing of random arrangements was associated with stronger activation in a fronto-parietal network of number related brain regions such as bilateral intraparietal sulcus and dorsolateral prefrontal cortex. This pattern of activation during visual quantification of unstructured arrangements closely replicates findings of previous studies, investigating the processing of non-symbolic numerical information (Piazza et al., 2004, 2007). Further, the involvement of areas associated with cognitive control comprising, amongst others, anterior and middle cingulate and dorsolateral prefrontal cortex (MacDonald et al., 2000) reflects that visual quantification of random arrangements required stronger implementation of control processes and performance monitoring.

In sum, whole brain results indicated different networks for processing structured and unstructured non-symbolic numerical information during estimation. Canonical and dice arrangements elicited activation in networks associated with the processing of familiarity, semantics and highly over-learned visual stimuli, reflecting the iconic properties of this structured stimulus material. In contrast, processing of random arrangements was reflected by the interplay of a brain networks associated with cognitive control and areas typically associated with magnitude processing, namely prefrontal cortex and bilateral IPS. Nevertheless, most areas constituting these two networks are not specific for number magnitude processing such as prefrontal or fusiform areas. Therefore, we specifically investigated the effect of set size and arrangement in intraparietal cortex, a region crucial for magnitude processing (e.g., Dehaene et al., 2003; Lyons et al., 2015), by means of ROI analysis.

### Neuronal Correlates of Subitizing and Estimation within IPS

We observed significant IPS activity during quantification of dot patterns within and above the subitizing range, with stronger activity in the estimation than the subitizing range. However, the amplitude of the neural response was modulated by numerosity in the estimation range only. An increase in numerosity in the subitizing range had no impact on the amplitude of the observed IPS response. This discontinuity in the slopes of neuronal response between subitizing and estimation range closely resembled the characteristic response pattern observed in the behavioral data. This seems to indicate a qualitative distinction between the processing of numerosity for small and large arrays of dots in bilateral IPS. To the best of our knowledge this is the first fMRI study that reports a signature in IPS response for a transition between subitizing and estimation processes. Importantly, our findings are in line with the results of a recent fNIRS study by Cutini et al. (2014) who reported that the hemodynamic response in the IPS as a function of numerosity was best fitted by a sigmoid function: for dot patterns exceeding the subitizing range, a steep increase in IPS response was observed followed by a tendency to plateau. However, Cutini et al. (2014) only presented dot patterns ranging from 2 to 6. Consequently, results regarding IPS response in the estimation range have to be interpreted with caution. Consistent with the findings of Cutini et al. (2014), we observed a steep increase in IPS response when the subitizing limit was exceeded. However, we did not observe an immediate tendency of the IPS response to plateau. Instead, IPS response increased linearly in the estimation range. This may be due to the fact that the estimation range in the present study was larger than in the study of Cutini et al. (2014) since participants enumerated dot patterns of up to 8 dots.

According to the abstract coding hypothesis of the ANS account, neurons in the IPS coding numerosity should be insensitive to the spatial arrangement of dots in a visual array (Dehaene et al., 1998; Dehaene et al., 2003; for a review see Cohen Kadosh and Walsh, 2009). To evaluate this, we presented dot patterns in random, canonical, and dice pattern arrangements. In the following the influence of the arrangement of elements in an array on IPS response will be discussed.

### Abstract vs. Format-Specific Representation of Numerosity

We extended the findings of Cutini et al. (2014) by demonstrating that the neural response in the IPS was also modulated by the spatial arrangement of dots in the arrays. Within the subitizing range the amplitude of IPS responses did not differ significantly between arrangements, whereas in the estimation range a clear differentiation of amplitudes was found. Random spatial arrangements led to strongest IPS activity, followed by canonical and dice arrangements. Interestingly, for dice arrangements the amplitude of IPS responses was similar in subitizing and estimation range. In fact, the slope of IPS activity as a function of numerosity was close to zero indicating that IPS activity did not increase with numerosity during quantification of dice patterns. In our view, this finding is of particular interest since it is hard to reconcile with the notion of abstract coding of numerical magnitude in the IPS (e.g., Dehaene et al., 1998). According to this account, neuronal populations in the IPS coding numerical quantity should be insensitive to the input format in which numerical information is presented (Dehaene et al., 2003). As a consequence, numerical information in different input formats (e.g., symbolic digits and non-symbolic dot patterns) should elicit a similar neural response in number sensitive IPS areas. However, the abstract coding account was recently challenged (Cohen Kadosh and Walsh, 2009). fMRI adaptation studies showed that recovery of IPS response after presentation of a deviant numerosity subsequent to repeated presentation of another numerosity in the adaptation phase was notation dependent (Cohen Kadosh et al., 2007). This challenges the assumption of an abstract representation of numerosity in the IPS (but see Piazza et al., 2007, for an alternative finding). Cohen Kadosh and Walsh (2009) pointed out that the amplitude of BOLD signal recovery after a deviant numerosity also interacts with the format of the numerical information and postulated format-specific representations of number magnitude.

In line with this notion, Lyons et al. (2015) found that although the IPS was involved during symbolic and non-symbolic number processing, activation patterns in the IPS differed fundamentally between notations. Applying RSA, they demonstrated that similarity curves of symbolic numbers had little to no overlap, whereas similarity curves of non-symbolic numerosities overlapped significantly, with increasing overlap as numerosities increased. The authors concluded that symbolic numbers are represented “in a more discrete fashion” whereas non-symbolic numbers are represented in “a more analog fashion” (Lyons et al., 2015, p. 484). This finding supports a notation dependent – and, therefore, non-abstract – quantity representation in the IPS.

In sum, previous studies indicated that non-abstract representations of numerical quantity exist that differ for symbolic and non-symbolic numerical information. Our study provides first evidence that even the quantity representation for non-symbolic numerical information might be (at least to some extent) format dependent and therefore non-abstract, because we observed that the amplitude of IPS activity was influenced by the spatial arrangement of dots during quantification. Backing the results of the PSC analysis, the RSA further revealed that the activation patterns in the IPS differed significantly between arrangements. The observed differences in the widths of similarity curves for dice, canonical, and random dot patterns speak against the idea of an abstract representation of non-symbolic numerosity (see also Cohen Kadosh and Walsh, 2009). Similarity curves for dice pattern were narrower than similarity curves for canonical arrangements, which in turn were narrower than similarity curves for random arrangements. Narrower similarity curves can be interpreted to reflect a more precise representation of numerosity in the IPS because the overlap between adjacent numbers decreases.

In particular, for dice patterns (and partly so for canonical arrangements) the precision of the underlying neural representation of quantity (as reflected by the width of the similarity curves) was not influenced by numerosity. This finding is in line with the results for the representation of symbolic numbers in a computational modeling study by Verguts and Fias (2004). After repeated coupling of non-symbolic and symbolic numerical input (e.g., “3” and “●●●”), numerosity-selective neurons that developed during unsupervised learning in a previously uncommitted neuronal network provided with non-symbolic stimuli also responded to the symbolic code. However, the overlap of tuning curves for symbolic input did not increase with increasing numerosity, indicating a more precise representation for this symbolic numerical input. Similarly, the width of tuning curves did not increase with increasing numerosity for dice patterns in the present study. This provides evidence that the numerosity of frequently perceived spatial arrangements (e.g., dice pattern and symmetrical arrangements) might be represented in a manner comparable to symbolic numerical input (e.g., Arabic digits) in the IPS.

### Quantification and Pattern Recognition

Another explanation for the difference between subitizing and estimation and the influence of arrangement on IPS activity might be the involvement of an additional mechanism that supports quantification and the formation of a precise representation of numerosity. We think that a pattern recognition process that particularly supports subitizing but also estimation of canonical and dice arrangements seems plausible (e.g., Mandler and Shebo, 1982). According to studies on pattern recognition, familiar configurations are recognized faster and less error-prone than random configurations of local elements. On the one hand, this would explain why we did not observe an effect of arrangement in the subitizing range. Three dots can often be perceived as a triangle and four dots as a square, irrespective of their spatial arrangement, making pattern recognition an efficient and reasonable process supporting visual quantification of small sets of objects. On the other hand, the distinct IPS response in the estimation range might also be accounted for by processes of pattern recognition. In case of canonical arrangements and dice patterns, the very same pattern recognition mechanism might be active, resulting in less activity but a more precise neural representation in the IPS (as indicated by the ROI and RSA results). Shorter RTs and reduced ERs for canonical and dice arrangements in the estimation range may be interpreted as support for the involvement of a pattern recognition mechanism. However, for random arrangements in the estimation range this mechanism may not be effective. Further fMRI studies are needed to revisit the question whether pattern recognition or a similar cognitive process (e.g., Gestalt perception, Wertheimer, 1923; Rennig et al., 2013) may explain why the IPS response is sensitive to the arrangement of dots during enumeration. Furthermore, it needs to be evaluated whether pattern recognition mechanisms can adequately explain the distinctive neural responses when processing small as compared to large visual arrays.

It has to be noted that increasing task difficulty was observed to be associated with stronger IPS activation itself. Because task difficulty increases with numerosity, the observed modulation of IPS response may simply reflect increasing task demands. However, various fMRI adaptation studies demonstrated that the IPS shows a number magnitude specific response even in passive viewing paradigms with no task demands (Eger et al., 2003; Piazza et al., 2004). Therefore, we are confident that IPS modulation in the present study reflected magnitude processing rather than mere difficulty effects. Moreover, the non-linearity of the IPS response at the transition between subitizing and estimation range cannot convincingly be explained by effects of task difficulty alone. In particular, the striking discontinuity in the IPS response around the subitizing limit violates the prediction of a linear increase of IPS response with increasing difficulty. Furthermore, numerosity for random and canonical arrangements was matched within the estimation range, meaning that dot patterns consisting of between 5 and 8 elements were presented in both conditions. Therefore, differences in IPS response between arrangements conditions cannot be explained by higher task difficulty due to an increase in numerosity alone. Therefore, we think that the observed modulation of IPS response as function of arrangement within the estimation range might reflect the impact of arrangement on non-symbolic magnitude processing. However, future studies should aim at further clarifying how the spatial arrangement of non-symbolic numerical information influences number processing.

## Conclusion

Taken together, our results suggest that the IPS is a key brain area for quantification processes both within *and* above the subitizing range (Piazza et al., 2002, 2004; Dehaene et al., 2003; Demeyere et al., 2014). However, the results of the ROI analysis showed a striking discontinuity in the amplitude of the IPS response between subitizing and estimation range, with a steep increase of activity for arrays with more than four elements. To our knowledge, this is the first fMRI study that found a signature in the IPS response for such a transition between subitizing and estimation processes. Furthermore, we observed that amplitude and pattern of IPS activation during enumeration depended on the arrangement of dots in the respective pattern. This is first evidence that even the representation of non-symbolic quantities in the IPS might not be abstract but format dependent (Cohen Kadosh and Walsh, 2009). In particular, our findings raise the question whether proposed models of non-symbolic magnitude representation (e.g., linear and logarithmic model; Pica et al., 2004) may only fit when the non-symbolic numerical input (e.g., dot patterns) is randomly arranged. We propose that dot patterns presented in a configuration frequently perceived (e.g., symmetrical or dice patterns) may even be represented similar to symbolic input. Therefore, the findings of the present study are hard to reconcile with the abstract coding of numerosity even for numerical information within a single notation. However, further studies with varying stimulus material are needed to further substantiate the idea of format dependent representations of numerical quantity within a single notation.

## Author Contributions

JoB: conceptualization, planning, data collection, data analysis, and writing the manuscript. JH: conceptualization, proofreading, and ANS expertise. EK: conceptualization and supporting data analysis. JuB: data collection and proofreading. JR: conceptualization, planning, data collection, and eye tracking. KM: conceptualization and proofreading. SH: conceptualization, supporting data analysis, and writing.

## Conflict of Interest Statement

The authors declare that the research was conducted in the absence of any commercial or financial relationships that could be construed as a potential conflict of interest.

## Figures and Tables

**FIGURE A1:**
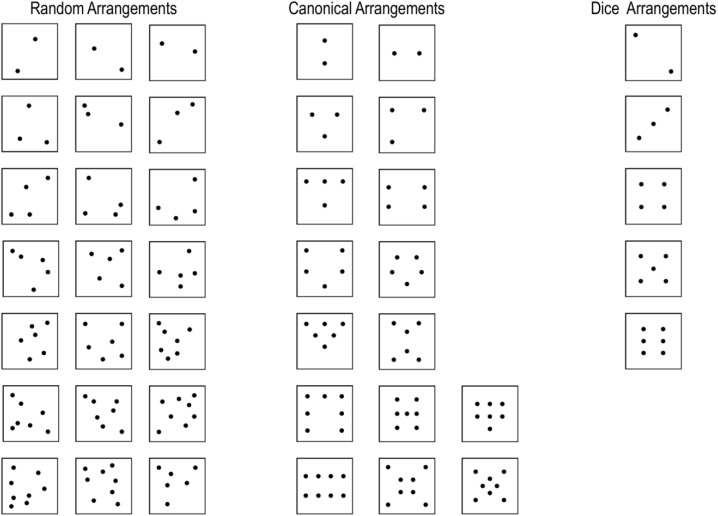
Dot pattern with random, canonical and dice arrangement adapted from Wender and Rothkegel (2000).

**FIGURE B1:**
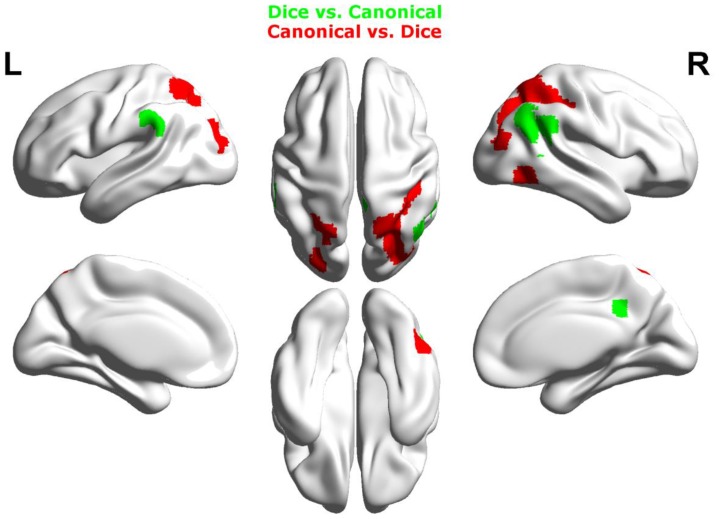
Results of the whole brain analysis. The contrast between dice and canonical arrangements within the estimation range (depicted in green) revealed activation in bilateral supramarginal gyrus as well as right hemispheric activation in angular gyrus and retrosplenial cortex. The reversed contrast canonical vs. dice within estimation ranged (depicted in red) revealed stronger activation in bilateral IPS as well as stonger activation in right inferior temporal gyrus and left middle occipital gyrus. Activation is depicted on a 3D rendered surface (all at *P*_cluster-corr_ < 0.001, cluster size of *k* = 15 voxels).

**FIGURE B2:**
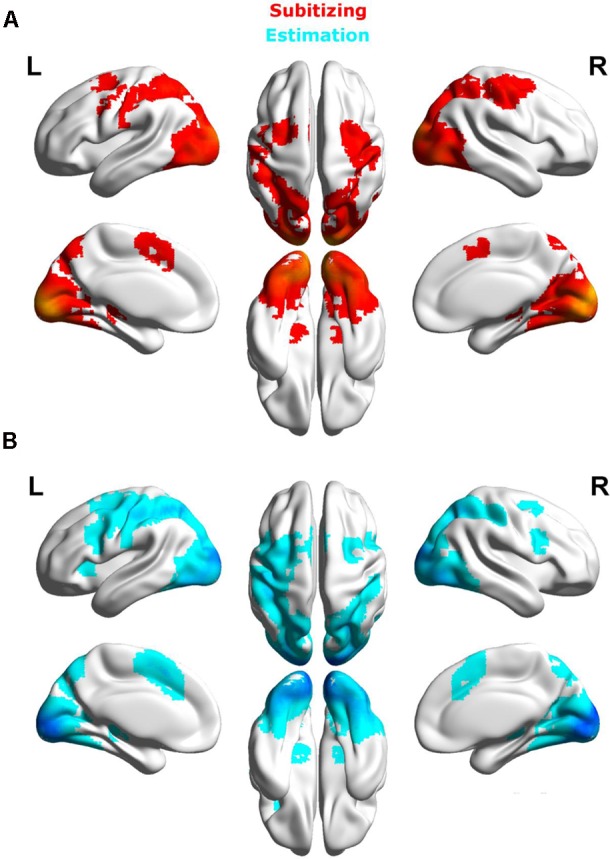
Results of the whole brain analysis. To provide a sanity check of our data, we provide simple contrasts against baseline. Both contrasts, Subitizing vs. baseline (**A**, depicted in red) as well as Estimation vs. Baseline (**B**, depicted in blue) reveald strong activation of a fronto parietal network (including bilateral IPS) and various visual and motor areas (see **Table B1** for details). Therefore, brain activation mirrors characteristics of the visual quantification task in which subsequent to the presentation of a visual stimulus a manual response was required. Activation is depicted on a 3D rendered surface (all at *p* < 0.05 (FWE) correction, *k* = 15 voxels).

## Footnotes

1 http://www.fil.ion.ucl.ac.uk/spm

2 http://marsbar.sourceforge.net

3 http://marsbar.sourceforge.net

## APPENDIX

**Table B1:** For sake of completeness we provide the remaining relevant contrasts between arrangements within the estimation range as well as simple contrasts against baseline.

Contrast	Brain region	MNI (x, y, z)			Cluster size	*T*
**Dice vs. canonical**						
	RH angular gyrus (PGa)	62	-54	23	271	4.91
	RH supramarginal gyrus (PF)^∗^	60	-54	35		4.92
	LH supramarginal gyrus (PF)	-66	-46	33	44	4.64
	RH retrosplenial cortex	7	-44	36	16	4.51
**Canonical vs. dice**						
	RH intraparietal sulcus (hIP1, hIP3)	32	-49	53	560	6.55
	LH intraparietal sulcus (hIP3)	-24	-63	56	76	5.93
	LH middle occipital gyrus	-28	-94	28	64	6.33
	RH inferior temporal gyrus	50	-64	-3	45	5.73
**Subitizing**						
	RH calcarine gyrus	10	-94	10	13504	30.01
	LH calcarine gyrus^∗^	-1	-91	3		27.63
	RH cuneus^∗^	12	-99	20		30.97
	LH cuneus^∗^	64	23	84		26.07
	LH thalamus parietal	-21	-31	0	198	11.12
	LH thalamus prefrontal^∗^	-13	-24	13		6.32
	RH thalamus visual	22	-31	0	136	9.70
	RH thalamus premotor^∗^	15	-21	10		6.06
	LH putamen	-21	9	8	67	6.27
	LH supplementary motor corex	-6	13	50	382	9.97
	LH middle frontal gyrus	-28	1	53	432	9.79
	LH precentral gyrus	-53	6	38	58	7.51
**Estimation**						
	LH posterior-medial frontal gyrus	-6	9	55	857	13.63
	LH anterior cingulate cortex^∗^	-11	24	33		6.34
	RH middle cingulate cortex^∗^	7	14	50		9.44
	RH superior frontal gyrus, extending into middle frontal gyrus	27	-4	58	243	9.71
	RH inferior frontal gyrus (p.opercularis)	47	4	30	150	8.71
	RH calcarine gyrus	12	-90	5	15799	29.03
	RH cuneus^∗^	12	-101	18		28.33
	LH superior occipital cortex^∗^	-13	-101	10		23.85
	LH middle occipital cortex^∗^	-18	-104	13		23.08
	Left supplementary motor cortex	-6	13	50	857	13.63
	LH thalamus parietal extending into thalamus prefrontal	-21	-27	-5	218	10.97
	RH thalamus	22	-31	0	46	9.13
	RH middle frontal gyrus	27	1	53	243	9.71
	RH precentral gyrus	47	8	25	150	8.71
	LH insular lobe	-31	21	5	86	7.89
	LH putamen	-21	13	3	148	7.86
	RH putamen	20	16	0	57	7.07

*^∗^Secondary peak. MNI, Montreal Neurological Institute; T, t-value; complex contrasts: *P*_*cluster-corr*_ < 0.001, cluster size of *k* = 15 voxels; simple contrasts: *P* < 0.05 (FWE) correction, cluster size of *k* = 15 voxels.*
